# Comparison of three generations of ActiGraph activity monitors under free-living conditions: do they provide comparable assessments of overall physical activity in 9-year old children?

**DOI:** 10.1186/2052-1847-6-26

**Published:** 2014-06-28

**Authors:** May Grydeland, Bjoerge Herman Hansen, Mathias Ried-Larsen, Elin Kolle, Sigmund Alfred Anderssen

**Affiliations:** 1 Department of Sports Medicine, Norwegian School of Sport Sciences, Oslo, Norway; 2 Institute of Sports Science and Clinical Biomechanics, University of Southern Denmark, Odense, Denmark

**Keywords:** Accelerometer, Physical activity, Children, Youth, Assessment

## Abstract

**Background:**

A recent review concludes that the agreement of data across ActiGraph accelerometer models for children and youth still is uncertain. The aim of this study was to evaluate the agreement of three generations of ActiGraph accelerometers in children in a free-living condition.

**Methods:**

Sixteen 9-year-olds wore the ActiGraph AM7164, GT1M and GT3X+ simultaneously for three consecutive days. We compared mean counts per minute (mcpm) and time spent at different intensities from the three generations of monitors, and the agreement of outputs were evaluated by intra-class correlation coefficients (ICC) and Bland-Altman plots.

**Results:**

The ICC for mcpm was 0.985 (95% CI = 0.898, 0.996). We found a relative difference of 11.6% and 9.8% between the AM7164 and the GT1M and AM7164 and the GT3X+, respectively. The relative difference between mcpm assessed by the GT1M and GT3X+ was 1.7%. The inter-generation differences varied in magnitude and direction across intensity levels, with the largest difference found in the highest intensities.

**Conclusion:**

We found that the ActiGraph model AM7164 yields higher outputs of mean physical activity intensity (mcpm) than the models GT1M and GT3X+ in children in free-living conditions. The generations GT1M and GT3X+ provided comparable outputs. The differences between the old and the newer monitors were more complex when investigating time spent at different intensities. Comparisons of data assessed by the AM7164 with data assessed by newer generations ActiGraphs should be done with caution.

## Background

In large scale studies, assessment of physical activity has shifted from self-reported estimates of activity levels to use of portable motion sensors, such as accelerometer-based activity monitors [1,2]. Activity monitors that contain an accelerometer provide accurate, reliable and feasible measurements of physical activity [2-4], and have recently been used in several nation-wide monitoring surveys of population levels of physical activity and temporal trends in physical activity [5-8]. Furthermore, recent intervention studies in children and adolescents aiming to increase physical activity have used activity monitors to estimate intervention effects [9-11]. Accurate and reproducible methods of measurements are vital in order for such estimates to be precise and independent of the known challenges related to self-reported physical activity (e.g. social desirability bias) and furthermore to make cross-cultural comparisons and to monitor effects of interventions aiming to increase physical activity.

In physical activity research, the ActiGraph (Pensacola, FL, USA) activity monitors are currently the most widely used accelerometer brand [12]. During the past decades, the ActiGraph monitors have been developed, with changes made to both hardware and firmware. The initial ActiGraph AM7164 has been replaced by newer generation monitors (i.e. GT1M, GT3X, GT3X+), with improvements in technological features, data storage capacity and researcher convenience. These improvements and changes in hardware provide some challenges related to the agreement between different generations of monitors, and have indeed been investigated [13-24]. While some studies report no difference between monitor generations [14,19,21,23,25], others urges for caution when comparing data assessed by different monitor generations due to differences in outputs [13,15,16,18,24,25]. The validation studies have mainly been performed in controlled settings (laboratory based or mechanical set up) leaving free living conditions less investigated. A recent review concludes that the agreement of data across ActiGraph models for youth still is uncertain [1]. We have earlier shown that the size and direction of bias between generations seem to depend on both movement intensity (e.g. frequency) and movement amplitude in a mechanical setup and free living in adults [18]. This suggests that the bias is highly dependent on the latent movement pattern. As the physical activity pattern is very different in children compared to adults (e.g. more intermittent in children) and differences between generations have been assessed in adults this warrants a comparison in children.

The aim of this study was to compare outputs from the three ActiGraph accelerometer generations AM7164, GT1M and GT3X+ when assessing physical activity among 9-year-old children in free-living conditions.

## Methods

### Participants

A sample of 36 children (mean age 9.9 y, SD = 0.3) were recruited to participate in the study. The participants were a randomly selected sub-cohort of an ongoing surveillance study (described elsewhere; [26]).

### Ethics statement

The children were informed about the study and parents provided written informed consent to participation. The study was reviewed by the Regional Committee for Medical Research Ethics and reported to the Norwegian Social Science Data Services.

### Instruments

Three generations of ActiGraphs activity monitors were used; model AM7164 (n = 18); GT1M (n = 18), and the GT3X+ (n = 18). These are small, robust and lightweight electronic devices that are attached to the body via elastic bands and assess movement.

### AM7164

The model AM7164 was launched in the 1990’s and discontinued in 2005 [27]. The AM7164 assesses acceleration by a built-in single-axis (vertical) piezoelectric accelerometer within a range magnitude of 0.05 – 2.13 *g*. The monitor has a filter that band limits the frequency range of the signals to a given range (0.21-2.28 Hertz). The AM7164 has a lower sampling frequency (10 Hertz) than the later generations and lower capacity with regards to memory and battery [14]. When 10 second epochs are used to assess activity, the AM7164 has a storage capacity of nearly four days. The AM7164 is initialized and downloaded over a serial port interface using a DOS-based program (RUI24, v. 2.13B, Computer Science and Applications INC., and monitor firm ware version 2.2 was used in this study).

### GT1M

The GT1M was launched in 2005 and replaced the AM7164. The GT1M (and all newer generations ActiGraphs) registers acceleration by a Micro-Electro-Mechanical System (MEMs) capacitive accelerometer. Like its predecessor, the GT1M has a filter that band limits the frequency range of the signals to a given range: 0.25-2.5 Hertz. The GT1M sampling frequency was set to 30 Hertz, and the monitor measures 0.05-2.5 *g* in dynamic range in the vertical axis. The GT1M and the newer monitor generations are initialized and downloaded over a USB interface using the ActiLife software (v. 5.5.5). In this study GT1M version 7.5.0 monitor firmware was used (produced in 2007). Detailed specifications of the GT1M are published elsewhere [27].

### GT3X+

In 2010, the GT3X+ was released with further improvements in technology. While the AM7164 and the GT1M samples data in user-determined time intervals (epochs), the GT3X+ has a configurable sampling frequency ranging from 30 – 100 Hertz that allows post-sampling filtering. The GT3X+ measures acceleration in a range of ±6 g. In addition to the vertical and medio-lateral axes, the GT3X+ measures a third, antero-posterior axis. In this study GT3X+ version 2.1.0 was used and sampling rate was set at 30 Hertz to match the sampling rate of the GT1M.

The AM7164 monitors were checked for calibration both before and after the comparison, using the Actigraph manufactured calibrator (Model CAL71) according the manufactures guidelines. Only activity monitors accepted by the calibrator were included in the study. The GT1M and GT3X+ units were exposed to a standardized set of sinusoidal accelerations in a mechanical setting, and tested for intra-instrument variation. The test and devices used are described in detail elsewhere [18]. In brief, the mechanical set up consists of two rotational wheels rotating in the vertical plane at a constant angular velocity. The wheels are connected with a connection rod (CR) and driven by an electric motor. The CR is attached away from the center of the rotational wheels. The monitors were firmly secured on a plate attached to the CR. This produces positive and negative accelerations in the vertical plane.

### Study protocol

The monitors were attached in triplets to a waist-worn elastic belt and mounted onto the children’s waist at the right hip (crest iliaca). The placement of each ActiGraph model was rotated and counterbalanced to avoid any potential order or placement effects. The monitors were initialized to assess activity in ten seconds epochs, and to start recording simultaneously. The children wore the monitors for three consecutive days, only removing them during water activities and while sleeping at night. Anthropometry was assessed objectively by trained staff. Weight and height were measured in light clothing and without shoes using standard procedures: Weight was measured in kilos (one decimal) using a digital Seca 877 scale (SECA GmbH, Hamburg, Germany). Height was measured in centimeters (one decimal) using a wall-mouthed measuring tape.

### Data reduction and statistics

Accelerometer data were processed by the custom made program Propero 1.0.17 (University of Southern Denmark). Since the hardware capacity of the AM7164 is limited to nearly four days at this epoch period, the accelerometer outputs from three days were used in the comparison between the different generations of ActiGraphs. Only daytime activity (06:00 – 24:00 hours) were included in the analyses. Sequences of 20 minutes or more of consecutive zero counts were interpreted to represent non-wear time and were excluded from each individual recording (to match the settings of the surveillance study). As monitor malfunctions and spurious data points are known to occur during field measurements [28], the outputs were checked manually. The total amount of physical activity from the activity monitor was expressed as the average of total counts per minute of registered time (counts/minute, cpm). Main outcome variable was mean count per minute (mcpm), an indicator of mean physical activity. Furthermore, minutes of intensity-specific physical activity were derived using the following cut-points: sedentary time <100 cpm, light physical activity 100 < 2000 cpm, moderate physical activity 2000 < 6000 and vigorous physical activity ≥6000 cpm. Moderate to vigorous physical activity (MVPA) was defined as ≥2000 cpm. These cut-points have been applied in earlier studies in this age group [26,29]. Between monitor-agreement was evaluated by calculating effect sizes based on pooled standard deviations and intra-class correlation coefficients (ICC) using a two-way mixed-model analysis of variance (ANOVA) with the assumption of absolute agreement. The differences based on the mean values of outcome were also expressed in percent. As the inter-monitor variability of the GT1M has been reduced compared to the AM7164 [16], outputs from the GT1M were used as reference to outputs from AM7164 and GT3X+. Bland-Altman plots were produced to investigate differences between the monitor outputs. All statistical analyses were performed using IBM SPSS (SPSS Inc., Chicago IL, USA) version 18 and a two-tailed alpha level of 0.05 was used for statistical significance.

## Results

A total of 16 participants provided data from all three monitors for at least 3 days and were included in the analyses. Of the 36 recruited participants, three volunteers did not show up for the study. Four of the AM7164 monitors malfunctioned during data-collection and resulted in data-loss from seven participants. Four AM7164 monitors lost calibration during data-collection which resulted in loss of an additional eight participants. Two participants had results that differed by more than 50% in mcpm from AM7164 to GT1M. These were defined as outliers and excluded from further analysis (see Additional file 1). The intra-instrument tests showed that all GT1M and GT3X+ monitors had a less than 1% variation, and we experienced no data loss from these monitors.

The analyzed sample (12 girls and 6 boys) had a mean age of 9.9 years (SD = 0.3), had a weight, height, and BMI of 35.1 kg (SD = 5.0), 139.6 cm (SD = 4.6), and 18.0 kg∙m^-2^ (SD = 2.2), respectively.

**Figure 1:**
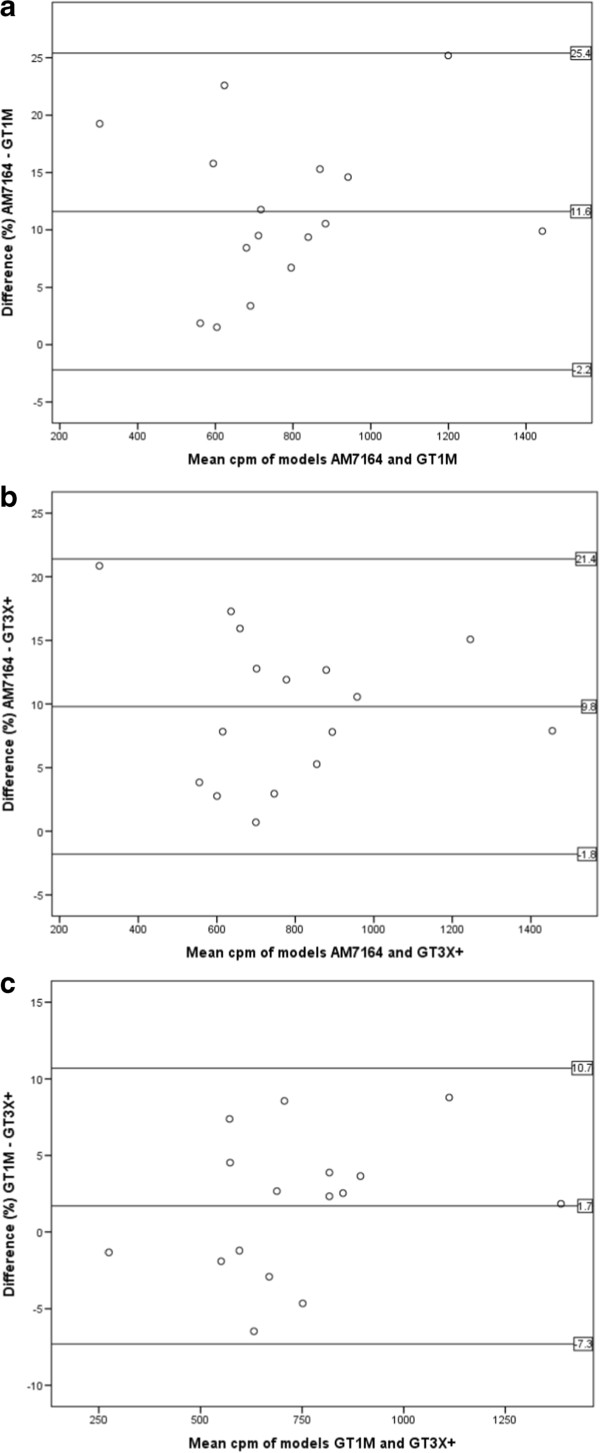
**Differences between 16 outputs of mcpm from AM7164 and a) GT1M and b) GT3X+ in percent, and c) difference between GT3X+ and GT1M in percent.** Reference-lines are mean differences and ±2SD.

Table 1 shows the mean values of physical activity outcome variables and valid wear time assessed by the different monitors. The ICC including the AM7164, GT1M and GT3X+ for mean physical activity (mcpm) was 0.985 (95% CI = 0.898, 0.996). The values of agreement between outputs from each monitor generation when assessing mcpm is shown in Table 2. All correlations were highly significant (p < 0.001).Figure 1 illustrates the agreement between the 16 recordings of mean physical activity (mcpm) by the different monitor generations in Bland-Altman plots.

**Table 1:** Outputs from the AM7164, GT1M and GT3X+ among 16 Norwegian 9-year-olds during 3 days of free-living data assessment (mean and SD)

***Mean (SD)***	**AM7164**		**GT1M**		**GT3X+**	
Wear time (accepted minutes/day)	767.7	(69.7)	759.7	(69.7)	761.9	(69.8)
Total physical activity (mcpm)	821.5	(287.3)	735.3	(245.1)	750.6	(260.4)
***Time (min/day) spent at intensities***						
Sedentary time <100 cpm	429.9	(72.0)	456.9	(72.8)	455.5	(75.6)
Light activity 100 < 2000 cpm	249.3	(41.5)	221.7	(48.9)	222.6	(45.5)
Moderate activity 2000 < 6000 cpm	71.5	(27.3)	69.4	(28.0)	71.7	(28.2)
Vigorous activity ≥6000 cpm	17.0	(9.1)	11.6	(7.6)	12.1	(8.0)
MVPA ≥2000 cpm	88.5	(33.0)	81.0	(30.2)	83.8	(31.3)

Mcpm: mean counts per minute, MVPA: moderate to vigorous physical activity.

**Table 2:** Agreement between outputs from AM7164, GT1M and GT3X+ in mcpm and time spent at different intensities (minutes/day) (n = 16)

	Mean difference in %* (95% CI)	Effect size**	**ICC (95% CI)**
**Total physical activity (mcpm)**			
Model 7164 – GT1M	11.7 (-4.0, 27.4)	0.32	.961 (0.368, 0.991)
Model 7164 - GT3X+	9.4 (-4.9, 23.7)	0.10	.977 (0.387, 0.995)
GT1M - GT3X+	2.1 (-4.9, 9.1)	0.06	.995 (0.984, 0.998)
**Sedentary time < 100 cpm**			
Model 7164 – GT1M	-5.9 (-17.4, 5.6)	0.37	.951 (0.189, 0.989)
Model 7164 - GT3X+	-5.6 (-16.7, 5.7)	0.35	.956 (0.289, 0.990)
GT1M - GT3X+	-0.3 (-3.0, 2.4)	0.02	.994 (0.982, 0.998)
**Light activity 100 < 2000 cpm**			
Model 7164 – GT1M	12.4 (-3.7, 28.5)	0.61	.880 (-0.105, 0.973)
Model 7164 - GT3X+	12.0 (-3.9, 27.9)	0.61	.865 (-0.060, 0.968)
GT1M - GT3X+	0.4 (-2.7, 3.5)	0.02	.993 (0.982, 0.998)
**Moderate activity 2000 < 6000 cpm**			
Model 7164 – GT1M	3.0 (-5.3, 11.4)	0.08	.979 (0.943, 0.993)
Model 7164 - GT3X+	-0.3 (-3.0, 2.4)	0.01	.989 (0.967, 0.996)
GT1M - GT3X+	3.3 (-5.5, 12.1)	0.01	.990 (0.971, 0.997)
**Vigorous activity ≥ 6000 cpm**			
Model 7164 – GT1M	46.6 (22.2, 71.0)	0.64	.876 (-0.133, 0.973)
Model 7164 - GT3X+	40.5 (16.4, 64.6)	0.57	.893 (-0.067, 0.976)
GT1M - GT3X+	4.3 (-5.6, 14.2)	0.06	.991 (0.973, 0.997)
**MVPA ≥ 2000 cpm**			
Model 7164 – GT1M	9.3 (-4.9, 23.5)	0.24	.968 (0.799, 0.991)
Model 7164 - GT3X+	5.6 (-5.7, 16.9)	0.15	.985 (0.929, 0.996)
GT1M - GT3X+	3.5 (-5.5, 12.5)	0.09	.991 (0.970, 0.997)

All correlations are significant at the 0.001 level. Mcpm: mean counts per minute, MVPA: moderate to vigorous physical activity. *Mean differences in percent are calculated based on the summed mean values. **Effect sizes are calculated based on pooled standard deviations.

When comparing minutes spent in different intensity levels the accelerometers showed diverging results (Table 2). With outputs from the GT1M as reference to the other monitor generations, negative relationships were seen for sedentary time, and positive relationships were seen for the higher intensities. The greatest divergence between monitors was observed in the vigorous intensity category: The relative difference between the AM7164 and the GT1M and GT3X+ amounted to 46.6% and 40.5%, respectively; according to the AM7164 the participants accumulated 17 minutes VPA while the GT1M and the GT3X+ showed an average of 11.6 and 12.1 minutes VPA per day, respectively.

## Discussion

The main finding of this study is that the ActiGraph model AM7164 yields higher outputs of mean physical activity (mcpm) than the ActiGraph models GT1M and GT3X+ in a free-living setting, while the generations GT1M and GT3X+ provide close to similar outputs. The differences between the old and the newer monitors were more complex when investigating time spent at different intensities.

Assuming that the GT1M and GT3X+ provide a more precise and stable mcpm output compared to the model 7164 [16], our findings show that data assessed by the AM7164 yield higher outputs of mcpm compared with the GT1M and GT3X+. This would indicate that physical activity levels assessed by the AM7164 could erroneously be interpreted as higher than physical activity levels assessed by the newer monitors. This could further have impact on public health policies and efforts to address a decreasing physical activity level based on methodological challenges rather than true observations.

The results from the intra-class correlations show almost perfect agreement. However, perfect agreement is the assumption for researchers using different monitor generations within studies and when comparing results across studies. Several validation studies including these monitors have been done over the last years and the conclusions vary. Most validations are done in mechanical setups or in a controlled laboratory settings [14-16,19,20,23,24,30], while a few have investigated the monitor outputs based on free living conditions [13,18,21,23,24,31]. One explanation for the varying conclusions can be that the results are population specific and different results can be expected dependent on age and activity type [18]. However, Reilly et al. [4] suggests that ActiGraph accelerometer outputs have little age- or size-related systematic variation for the same behavioral input across a wide age/size range (3–10 years). Cain et al. [1] state that there is growing evidence of differences in sensitivity of ActiGraph accelerometers outputs among adults, and that it is unclear how model differences affect interpretation of data from children. Our results support the limited cluster of research stating that there is a difference between the old AM7164 and the newer ActiGraph models, and that these findings might affect interpretation of accelerometer data obtained from children and adolescents [13,25]. Our results also support the growing number of studies showing that data assessed by the newer generation ActiGraphs, from GT1M and forward, can be compared and used interchangeably [18,19,21,23].

The observed differences varied in magnitude across intensity-levels. The largest differences were seen at the highest intensities, where the children spent the least amount of time (less than 1% of the measured time). As both size and direction of the inter-generation differences were intensity dependent, the absolute difference would depend on time spent at the certain intensities, and the cut-points applied. Furthermore, the epoch length also appears to affect the outcome when comparing outcomes of different monitors [13], as well as how a valid day is defined with regards to the definition and subsequent handling of spurious data and periods of non-wear [28].

Some authors have suggested applying a correction factor to data obtained by one of the monitor generations to correct for this difference, for mean physical activity (mcpm). Corder et al. [2] suggested multiplying the data derived from the AM7164 with 0.91 in order to make data comparable with data from GT1M. The corresponding correction factor in this study would be to multiply data assessed by the AM7164 with 0.88 to be comparable to GT1M-data for mcpm, and 0.90 to be comparable to GT3X+ -data for mcpm, based on the relative differences in mcpm of 11.6% and 9.8%, respectively. As the inter-generation difference in mcpm varied across intensities, we acknowledge that correcting the mcpm might introduce an unknown bias. We do not know the size of bias caused by frequency and amplitude. The suggested correction factors would only apply to similar distributions of time spent across intensities. The results of this study might imply that intensity-specific cut-points should be generation specific. However, in order to provide such recommendations, the study needs to be repeated in larger samples. Based on these considerations we did not find it appropriate to suggest intensity-specific correction factors to aid the demonstrated divergence. However, we urge for caution when comparing intensity-specific data assessed by AM7164 with newer generation ActiGraph accelerometers.

As the AM7164 was discontinued in the mid 2000s, the chances of this model being used in future assessments are small. However, we worry that future studies will attempt to compare data across studies including data from the AM7164 and compare historical data with newer data to elucidate trends over time. Such comparisons across ActiGraph generations, including the AM7164, should be done cautiously.

### Strengths and limitations

The strength of this study was the multiple accelerometers worn simultaneously of children in a free living condition. However, there are some limitations. The sample size was small and we experienced a relatively large drop out due to incomplete data. However, despite the small sample we observed significant differences between monitors. Furthermore, we did test our hypothesis in a mechanical setup and a second free living study (n = 20 adults) [18]. The main findings that AM7164 gives different and higher total mcpm than the newer models confirm our results showing that physical activity assessed by the AM7164 should be treated with caution in comparison with data assessed by the newer generations of ActiGraphs.

The study comprises 9-year-old children only, and this hampers the generalizability of the results to other populations such as adults. Furthermore, as multiple settings exist (regarding epoch length, definition of valid days, non-wear time, intensity cut-points etc.) this limits the generalizability of these findings to apply to other settings.

## Conclusion

We found a significant difference between the older ActiGraph accelerometer AM7164 and the newer generations GT1M and GT3X+ when assessing mean physical activity among 9-year-olds in a free living condition. The results suggest that data assessed by AM7164 should not be compared to newer generation ActiGraph accelerometers without careful considerations. There were no significant differences in mcpm between the GT1M and GT3X+. Comparisons of intensity-specific physical activity data assessed with the AM7164 and newer accelerometer generations should be done with caution as the differences are not systematic.

## Competing interests

The authors declare that they have no competing interests.

## Authors’ contributions

MG: participated in conceptualizing the study, coordination of data collection, conducted analyses, drafted the manuscript, and made the greatest contribution to the manuscript. BHH: participated in conceptualizing the study, reviewed and revised the manuscript, and approved the final manuscript. MRL: participated in conceptualizing the study, reviewed and edited the manuscript, and approved the final manuscript. EK: participated in study coordination and data collection, reviewed and revised the manuscript, and approved the final manuscript. SAA: participated in conceptualizing the study, reviewed and revised the manuscript, and approved the final manuscript as submitted. All authors read and approved the final manuscript.

## Pre-publication history

The pre-publication history for this paper can be accessed here:

http://www.biomedcentral.com/2052-1847/6/26/prepub

## Supplementary Material

Additional file 1 **Box-plot of total sample (n=18) showing difference between AM7164 and GT1M outputs in mcpm (%).** With a median difference of 11.7% between monitors case number 10 and 11 are extreme outliers showing about 50% difference between AM7164 and GT1M outputs of total physical activity. Data from these cases were therefore disregarded. **Figure S1.** Box-plot of total sample (n=18) showing difference between AM7164 and GT1M outputs in mcpm (%). Case number 10 and 11 are defined as extreme outliers. Click here for file
