# Supplementary material for: Comparison of three generations of ActiGraph activity monitors under free-living conditions: do they provide comparable assessments of overall physical activity in 9-year old children?
Source: BMC Sports Sci Med Rehabil. 2014 Jun 28;6:26. doi: 10.1186/2052-1847-6-26 (PMC4100529; doi:10.1186/2052-1847-6-26)
Supplement: Additional file 1 — Box-plot of total sample (n=18) showing difference between AM7164 and GT1M outputs in mcpm (%). With a median difference of 11.7% between monitors case number 10 and 11 are extreme outliers showing about 50% difference between AM7164 and GT1M outputs of total physical activity. Data from these cases were therefore disregarded. Figure S1. Box-plot of total sample (n=18) showing difference between AM7164 and GT1M outputs in mcpm (%). Case number 10 and 11 are defined as extreme outliers. [file 2052-1847-6-26-S1.docx]

**Additional file**

Box-plot of total sample (n=18) showing difference between AM7164 and GT1M outputs in mcpm (%). With a median difference of 11.7% between monitors case number 10 and 11 are extreme outliers showing about 50% difference between AM7164 and GT1M outputs of total physical activity. Data from these cases were therefore disregarded.


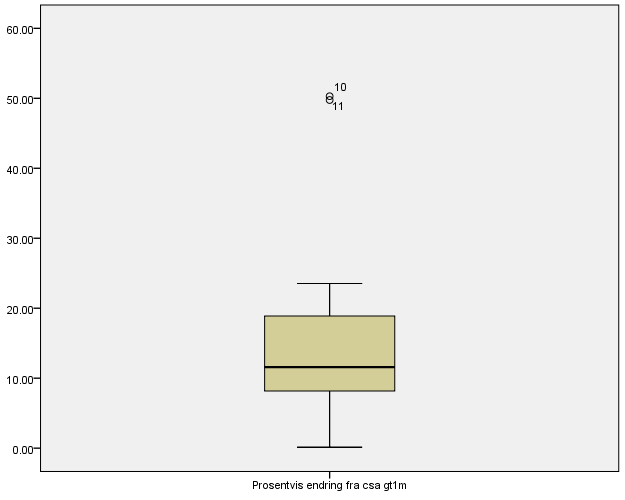


Difference between AM7164 and GT1M in mcpm

%

**Figure 1** Box-plot of total sample (n=18) showing difference between AM7164 and

GT1M outputs in mcpm (%). Case number 10 and 11 are defined as extreme outliers.
